# Antarctic Marine Biodiversity and Deep-Sea Hydrothermal Vents

**DOI:** 10.1371/journal.pbio.1001232

**Published:** 2012-01-03

**Authors:** Steven L. Chown

**Affiliations:** Centre for Invasion Biology, Stellenbosch University, Stellenbosch, South Africa

## Abstract

The diversity of many marine benthic groups is unlike that of most other taxa. Rather than declining from the tropics to the poles, much of the benthos shows high diversity in the Southern Ocean. Moreover, many species are unique to the Antarctic region. Recent work has shown that this is also true of the communities of Antarctic deep-sea hydrothermal vents. Vent ecosystems have been documented from many sites across the globe, associated with the thermally and chemically variable habitats found around these, typically high temperature, streams that are rich in reduced compounds and polymetallic sulphides. The animal communities of the East Scotia Ridge vent ecosystems are very different to those elsewhere, though the microbiota, which form the basis of vent food webs, show less differentiation. Much of the biological significance of deep-sea hydrothermal vents lies in their biodiversity, the diverse biochemistry of their bacteria, the remarkable symbioses among many of the marine animals and these bacteria, and the prospects that investigations of these systems hold for understanding the conditions that may have led to the first appearance of life. The discovery of diverse and unusual Antarctic hydrothermal vent ecosystems provides opportunities for new understanding in these fields. Moreover, the Antarctic vents south of 60°S benefit from automatic conservation under the Convention on the Conservation of Antarctic Marine Living Resources and the Antarctic Treaty. Other deep-sea hydrothermal vents located in international waters are not protected and may be threatened by growing interests in deep-sea mining.

## Abstract 2

A recent study has demonstrated that deep-sea hydrothermal vents in the Antarctic have rich and unusual animal communities. This discovery highlights the importance of the Antarctic benthos for biological understanding.

For most groups of organisms diversity is highest in the tropics. A hectare of rainforest contains more plant species than a similar area of Norwegian boreal forest. Likewise, on average, tropical latitudes are home to more fish, bird, and mammal species than anywhere else [1]–[3]. Whilst this foremost pattern of biodiversity has long been known, its nuances and the exceptions to it are less widely appreciated. For example, in terrestrial birds and ants the decline in diversity on either side of the equator is not symmetric. It is steeper in the north [4],[5], reflecting a broader suite of biological differences among the hemispheres [6]. Perhaps more striking is the far wider variety of biodiversity gradients found in marine groups, and the almost total contrast in many benthic (bottom-dwelling) taxa to the typical terrestrial latitudinal diversity gradient.

For coastal marine species, such as fish or corals, richness tends to be highest in the tropics, especially the Indo-West Pacific, whilst for more ocean-going, or pelagic species, such as the albatrosses and petrels, diversity peaks are typical of the mid-latitudes [7],[8]. In both cases, diversity still tends to drop off at the sub-polar and polar latitudes. In many benthic groups the pattern is completely different. Diversity does decline in the far north, but does not do so in the Southern Ocean (the region around and to the south of the Antarctic Polar Front—the dynamic location where cold Antarctic and warmer sub-Antarctic waters meet). Rather, some groups show their highest richness here. Taking survey effort and area into account, organisms such as pycnogonids, bryozoans, isopods, amphipods, sponges, ascidians, and echinoderms are remarkably diverse in the Southern Ocean, as much or more so than is typical of many, more equatorial locations [9],[10]. Recent, large-scale surveys, conducted, in part, under the auspices of the international Census of Marine Life have borne out these patterns [11]–[13], highlighting the extraordinary diversity of the Antarctic [14]. Moreover, they have also drawn attention to the considerable endemism, i.e., restriction to a given area (in this case the Antarctic), of much of the benthos.

The unique occurrence of species in certain areas reflects a general pattern of diversity found across the planet. As one travels away from a given point, so the environment tends to differ progressively and, as a consequence, the identity of species also changes. The greater similarity of points that are close to each other than those that are more distant is known as spatial autocorrelation [15], and may well lie at the heart of many of the most fundamental and familiar patterns in the distribution and abundance of organisms [16]. The change in species identity through space can be reflected mathematically using alpha-diversity—a measure of local diversity, beta-diversity—a measure of turnover among closely located sites, and by gamma-diversity—the diversity of the landscape [17]. Over relatively short distances beta-diversity reflects a suite of biological processes, such as environmental tolerances, and interspecific interactions, such as competition or predation, which lead to species replacement. At the much larger, gamma-diversity scale, the role of evolutionary history and its interaction with the geological evolution of a region tends to predominate, so often leading to wholly different biotas among regions, and endemism at high taxonomic levels [18]. More simply put, within Australia, one tends to find different species of wallabies and kangaroos replacing each other as one drives from Hobart to Darwin, whereas when driving from Cape Town to Dar es Salaam (a similar distance) the turnover would be among antelope, given that marsupials are entirely absent from Africa. Naturally, there's some interaction among the regional and local scales, so accounting for variation in biodiversity patterns across the planet, such as the anomalously high richness and endemicity of plants at the south-western tip of Africa [3].

In a similar way, a notable feature of the Antarctic benthos is that endemism is generally high compared with other regions. In other words, the fauna is not only rich, but it is most unusual too [14]. In this issue of *PLoS Biology*, Rogers et al. [19] show that this is true also of the animals associated with deep-sea hydrothermal vents, providing the first comprehensive assessment of such ecosystems in the Antarctic. Although the presence of deep-sea hydrothermal vents in the region had previously been recorded, they had neither been comprehensively explored, nor thought to be home to a diverse vent fauna [20],[21]. This is not entirely surprising. These vents are located thousands of metres below the ocean surface and are challenging to explore. Detection and verification of deep-sea hydrothermal vents require a combination of geophysical and biological data. The former are typically obtained using echo sounders, optical light-scattering sensors mounted on instruments towed by research vessels, and conductivity, temperature, and depth (or CTD) instruments, which enable water sampling at different depths. Further exploration and sampling of sites identified using these methods is then undertaken either by remotely operated vehicles (ROVs) or deep water submersibles, in combination with the geophysical approaches.

Hydrothermal vents are formed by plumes of heated water associated with plate boundaries, typically divergent boundaries such as mid-ocean ridges, but also the more complex, back-arc basins associated with subduction zones [22],[23], and their unusual ecosystems were first documented in the late 1970s [24]. Since then the image of “black smokers” surrounded by hundreds of thermophilic tubeworms and other strange animals has become part of the modern, natural history psyche. However, this image is somewhat misleading. In the same way that diversity varies locally and regionally in terrestrial systems, so too do the communities of deep-sea hydrothermal vents vary across the globe [25]. In other words, a distinct biogeography of vent ecosystems exists (Figure 1). Different communities of organisms are characteristic of hydrothermal vents in various parts of the world as a consequence of differences among ocean basins, rates of seafloor spreading, local turnover, and variation in the life histories and dispersal characteristics of the organisms involved [26]–[29].

**Figure 1:**
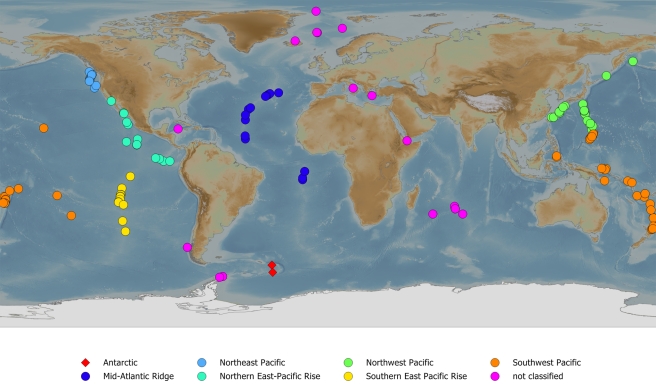
Deep-sea hydrothermal vent systems that require conservation. At the request of the International Seabed Authority, InterRidge (a non-profit organization, promoting mid-ocean ridge research, see http://www.interridge.org/about) advised that the vents shown here should be protected from exploitation, though with some InterRidge members arguing that all sites should be protected (http://www.interridge.org/node/1, see also Van Dover [37]). Vent biogeographic provinces identified by Bachraty et al. [28] are indicated in colour, and the two East Scotia Ridge Sites described by Rogers et al. [19] are indicated with diamonds, just to the east of the Antarctic Peninsula. A full list of vent sites can be found on InterRidge's pages at: http://www.interridge.org/IRvents. The base map is the NOAA global relief model (http://www.ngdc.noaa.gov/mgg/global/global.html). Figure compiled by Aleks Terauds.

What makes the findings of Rogers et al. [19] so remarkable is that the communities of these vents, located at c. 56°S to 60°S along the East Scotia Ridge (ESR) (Figure 1), are unlike those anywhere else. For example, hydrothermal vents along the East Pacific Rise and Galápagos Rift are dominated by siboglinid tubeworms (mainly *Riftia pachyptila*), bathymodiolid mussels, alvinellid polychaetes, and vesicomyid clams. Along the northern Mid-Atlantic Ridge, alvinocaridid shrimp (mostly *Rimicaris exoculata*) and bathymodiolid mussels are most common [25],[28]. The East Scotia Ridge vent sites are dominated by dense aggregations of a new species of yeti crab (*Kiwa* sp., so called because of the dense arrangements of the setae on the ventral surface, or on the pincers), a new species of peltospiroid gastropod, a previously unknown *Vulcanolepas* stalked barnacle, several anemone species, and a predatory sea star. Taxa typically common at other hydrothermal vents, such as alvinocaridid shrimp, polychaete worms, and bathymodiolid mussels are absent [19].

Importantly, Rogers et al.'s [19] findings not only emphasize the unique nature of Antarctic biodiversity in a global context, but also draw attention to the fact that deep-sea hydrothermal vent communities show much more inter-regional diversity generally than has been found to date. The significance thereof lies especially with the microbiota that exploit these environments. Hydrothermal vents are found where sea water enters the underlying rock through cracks caused by ocean floor spreading, is then heated, and ejected back into the water mass. Typically, the main vent streams reach high temperatures (as much as 400°C), have acidic pH values of 2 to 3, and contain a range of metals and reduced compounds, such as hydrogen sulphide [22],[30]. Because of the low temperature of the deep sea (c. 2°C, and 0°C to −1.3°C in the case of the ESR vents), conditions are highly variable over very small distances in the proximity of vents, making the environment patchy, both thermally and chemically, so providing a rich diversity of habitats for archaea and bacteria. The only exception to the general pattern found so far has been the off-axis vents of the Mid-Atlantic Ridge Lost City site. They are characterized by lower temperatures (<90°C), alkaline streams (pH 9–11), and a process known as serpentinization [31], and may, in consequence, provide unique insights into life's origins [30]. At the typical, black smoker–type deep-sea vents, many of the bacteria fuel primary production by obtaining energy from the oxidization of reduced sulphur compounds or methane [22],[30],[32]. The fact that bacteria are capable of doing so may not come entirely as a surprise, given their remarkable biochemical diversity. Rather, it is the metazoans that have proven extraordinary. When first examined, it was assumed that the animals occurring at the vents were heterotrophs, grazing on the abundant microbiota found at the sites. This is indeed one way, along with filter-feeding, that vent metazoans power their lifestyles [22],[27]. However, in other vent animals, groups of bacteria are found as chemosynthetic endosymbionts (i.e., internally located) or episymbionts (located externally) [32]. Thus, these metazoans do not obtain their nutrients in a way that would be familiar to us, but rather derive all of their nutrition from the chemosynthetic symbionts. For example, the tubeworm *R. pachyptila* lacks a digestive system and depends entirely on its endosymbiotic bacteria housed in a special organ known as the trophosome. Other groups of organisms dependent on chemosynthetic symbionts include bivalve and gastropod molluscs, and shrimp and crabs.

Although the bacteria examined by Rogers et al. [19] show high genetic sequence similarity to others typical of hydrothermal vent sites, the possibility of new forms of chemosynthetic relationships is highlighted by the presence of a variety of new vent metazoan species that may play host to such bacteria. Further exploration of these kinds of relationships, in newly characterized vent sites such as the ESR, might well reveal additional biochemical diversity, providing compelling reasons for on-going investigations. The benefits of such research are clearly illustrated by the recent discovery of hydrogen oxidation (so eclipsing human pretensions to the first hydrogen economy) in symbionts of *Bathymodiolus* mussels from the Mid-Atlantic Ridge, and probably other vent organisms (*R. pachyptila* and the shrimp *R. exoculata*) [33]. Several other redox reactions are plausible for, but as yet undiscovered in, vent systems [22].

The hydrothermal vent communities found at ESR site E9 differ in a further, significant way to most others. They are protected by the Convention on the Conservation of Antarctic Marine Living Resources (CCAMLR) (http://www.ccamlr.org), and by the Antarctic Treaty [34]. Nonetheless, both sites E2 and E9 are included within the Exclusive Economic Zone claims of the UK and Argentina [35]. Whilst sites north of 60°S remain the subject of dispute, competing claims for areas south of 60°S are essentially frozen under the Antarctic Treaty [34]. Although CCAMLR includes rational use, it was established within the Antarctic Treaty System specifically for the conservation of Antarctic marine ecosystems. While several hydrothermal vent systems elsewhere are included within marine protected areas, those in international waters enjoy no such conservation status [36]. Why should such protection be important for systems located so far below surface waters? In addition to their extraordinary ecosystems, deep-sea hydrothermal vents are characterized by an abundance of metal sulphides that precipitate out from the black smokers [37]. At the Solwara Vent site off the coast of Papua New Guinea, deposits are thought to contain c. 7% copper by weight compared with levels a tenth as much found in copper mines on land [38]. In consequence, Nautilus Minerals of Canada intends to start mining these deposits in 2013. Although the environmental impacts at this particular site will, apparently, be well managed, interest in mining deep-sea hydrothermal vents is likely to increase [37]. Indeed, the International Seabed Authority (http://www.isa.org.jm/) approved, in July this year, four new applications for exploration of polymetallic sulphides associated with hydrothermal vents. The applicants will restrict activity to inactive sites, which do not play host to typical, living vent communities [29], but much concern has been expressed about the potential for damage to sites in international waters in the absence of an agreed and effective conservation policy (Figure 1) [37],[38]. Other potential conservation and governance challenges include increasing interest in natural products from vent ecosystems [39], while scientific exploration brings its own concerns. However, the latter are well recognized [40], and research is typically careful, as demonstrated by the exploration of the ESR vents [19]. Nonetheless, for the ESR E9 and other Antarctic hydrothermal vents, the Antarctic Treaty System brings the advantage of a long history of well-developed practices to restrict the impacts of human activities. In particular, the Protocol on Environmental Protection to the Antarctic Treaty currently prevents any activity relating to mineral resources, other than scientific research. Whilst the Treaty System is by no means flawless in its regulatory responsibilities, to date it has achieved a far greater degree of stewardship of Antarctica and the Southern Ocean than is typical of many other commons [34].
